# Using multiple linear regression to predict engine oil life

**DOI:** 10.1038/s41598-025-18745-w

**Published:** 2025-09-29

**Authors:** Van Thai Nguyen, Jan Furch, Jan Koláček

**Affiliations:** 1 Faculty of Military Technology, University of Defence, 65, Kounicova 65, Brno, 66210 Czech Republic; 2 Faculty of Science, Masaryk University, Kotlářská 2, Brno, 63700 Czech Republic

**Keywords:** Engineering, Materials science

## Abstract

This paper deals with the use of multiple linear regression to predict the viscosity of engine oil at 100 °C based on the analysis of selected parameters obtained by Fourier transform infrared spectroscopy (FTIR). The spectral range (4000–650 cm⁻¹), resolution (4 cm⁻¹), and key pre-processing steps such as baseline correction, normalization, and noise filtering applied prior to modeling. A standardized laboratory method was used to analyze 221 samples of used motor oils. The prediction model was built based on the values of Total Base Number (TBN), fuel content, oxidation, sulphation and Anti-wear Particles (APP). Given the large number of potential predictors, stepwise regression was first used to select relevant variables, followed by Bayesian Model Averaging (BMA) to optimize model selection. Based on these methods, a regression relationship was developed for the prediction of viscosity at 100 °C. The calibration model was subsequently validated, and its accuracy was determined using the Root Mean Squared Error (RMSE) metric, it was 0.287. Finally, the obtained model was used to predict the lifetime of engine oil in diesel engines operating under severe conditions.

## Introduction

Viscosity is one of the most important physical properties of engine oil. Viscosity measurement is the first basic and mandatory step in oil analysis and is considered by oil and engine manufacturers as a key indicator of lubrication quality. It plays an important role in determining the lubricant flow rate through an internal combustion engine, thus establishing a fundamental balance for lubricant performance^1,2^. During lubrication with engine oil, a thin film is formed on the surface between the moving metal parts, which reduces friction and wear of the lubricated parts [3]. 

Lubricants with the appropriate viscosity can withstand high loads and protect metal surfaces from direct contact, especially under severe operating conditions.

Modern engine oils contain additives that are designed for the operation of powerful internal combustion engines and at the same time extend the life of these oils^4^. These additives play a vital role in maintaining low friction and reducing wear, especially under high load conditions, such as anti-wear agents, corrosion inhibitors and antioxidants^5^. Over time, these additives are consumed or degrade, leading to a reduction in their effectiveness. Depletion of these additives can result in increased oil acidity^6^.

Lubricating oil contamination is the main cause of engine wear^7,8^. Contaminants such as water, dust, or chemicals can enter the lubricant and cause chemical reactions that accelerate oil degradation by increasing its acidity. Acidic contamination is common in machines exposed to harsh environments or where contact with other fluids is possible^9^.

Oxidation occurs when the lubricant reacts with oxygen, usually at high temperatures. This reaction leads to the formation of acidic by-products, such as carboxylic acids and peroxides, which raise the acid number of the oil. Oxidation is therefore one of the primary mechanisms of engine oil degradation at elevated temperatures^10^.

Factors such as an increase in wear particles, soot contamination, or the use of higher-viscosity lubricants lead to a rise in lubricant viscosity^11^. Conversely, dilution by fuel or water, the use of lower-viscosity oils, or viscosity index improvers in multigrade lubricants may decrease viscosity. This reduction impairs the oil’s ability to form a protective film between moving parts, leading to higher friction, accelerated wear, and potential equipment failure^10,12^.

Viscosity, viscosity index (VI), total base number (TBN) and total acid number (TAN) are key parameters for evaluating the quality of engine oil and determining the optimum drain interval^13^. Fourier transform infrared spectroscopy (FTIR) is nowadays an accepted and widely used tool for analyzing the composition and monitoring the condition of oil. With advanced software using multivariate statistical algorithms, researchers are developing models to predict various quality parameters of crude oils, lubricants and fuels^14^. Predictive models based on FTIR spectra allow estimation of TBN and TAN values as an alternative to traditional potentiometric titration, which is time consuming and requires manipulation of chemical reagents. However, for accurate viscosity index determination, it is necessary to combine FTIR with other viscosity measurement methods^1,15–17^. Various predictive models are used to determine the total acid number (TAN), including Projection Pursuit Regression (PPR), Partial Least Squares (PLS), Support Vector Machines (SVM), linear models and Random Forest. In the study, the PPR model achieved a 37% reduction in root mean square error (RMSE) and 51% reduction in mean absolute error (MAE) compared to the general linear model (GLM). The RMSE and MAE values in model validation were 0.759 and 0.359, respectively^18^. The most common multivariate methods for spectral region analysis include principal component analysis (PCA)^19^ partial least square (PLS)^20^ interval PLS (iPLS), and principal component regression (PCR)^21^.

In recent years, numerous studies have successfully applied machine learning algorithms and statistical models to predict the physicochemical properties of complex liquids. For instance, neural networks have been utilized to forecast the fuel consumption of marine engines^22^ while multilayer perceptron models have demonstrated high effectiveness in simulating thermal systems^23^. In addition, models that combine ensemble machine learning techniques with the SHAP interpretability method have achieved high accuracy in predicting biochar yield, while also providing transparent insights into the importance of each input variable^24^.

Currently, the following methods are used to predict viscosity, such as empirical models that use experimentally obtained data to establish relationships between viscosity and factors such as temperature, pressure, and engine oil contamination.

A technical paper^25^ discusses empirical correlations of lubricant viscosity. Another applicable method is machine learning and the use of artificial neural networks. These modern approaches involve the use of machine learning algorithms to model complex nonlinear relationships affecting viscosity. A study^26^ demonstrates the use of neural networks to predict the viscosity of biodiesel blends, which is relevant to understanding the application of machine learning in this field. In study^27^ a physically informed machine learning model capable of predicting the temperature dependence of the viscosity of oxide liquids is presented. The study^28^ provides valuable insights for the optimization and implementation of machine learning models in predicting the viscosity of engine lubricants. Limitations include the size of the dataset, which may affect the generalization of the findings and the omission of other factors affecting engine performance. However, this study lays the foundation for future research on the application of soft computing tools in engine oil analysis and condition monitoring. Another method used is molecular dynamic simulation. This method simulates the behaviors of molecules in the oil at the atomic level, allowing viscosity to be predicted based on molecular composition. Paper^29^ presents results from which reliable estimates of viscosities of mixtures of industrially relevant ester-based lubricants at different temperatures can be obtained. Finally, combined approaches can be used. Some studies combine multiple methods, such as empirical models with neural networks, to increase the prediction accuracy^26^. These methods provide different levels of accuracy and complexity, with the selection of the appropriate method depending on the specific application and the available data. The above study^30^ demonstrated the successful prediction of biodiesel properties (e.g. viscosity) using FTIR spectra combined with multiple linear regression and artificial neural networks, the performance of which is comparable to that of standard PLS regression with full spectrum. The obtained results show that the proposed approach is simple, reliable and straightforward and provides better prediction with average absolute percentage error of 4.62%, 1.04%, 2.75% and 6.85% for kinematic viscosity, density, higher heating value and cetane number of biodiesel.

Currently, both predictive and proactive maintenance strategies based on engine oil analysis combined with data analysis are essential to support preventive maintenance decisions^31,32^. By monitoring the system condition and degradation using diagnostic signals and modelling the technical degradation of the system condition, it is possible to estimate the remaining useful life (RUL) of the system under investigation. This approach also provides a number of benefits, such as reducing maintenance costs, minimizing downtime, extending machine life and optimizing life cycle costs^33,34^.

## Research methods and material

### Description of the experiment

As part of the experiment, 221 samples of worn Mannol Multifarm STOU 10 W-30 engine oil were taken from 18 heavy crawler vehicles using a Perkins CV 12-1000 TCA diesel internal combustion engine rated at 746 kW. The sampling was carried out according to the technological procedure described in^2^. The samples collected operating under varying load and environmental conditions. Out of the total, 205 samples were used for model training, while 16 separate samples were reserved for model testing to evaluate its predictive performance.

Mannol Multifarm STOU 10 W-30 is a highly versatile engine and gear oil designed for modern heavy duty wheeled and tracked equipment powered by diesel engines, both turbocharged and non-turbocharged. It is a semi-synthetic oil that provides maximum engine protection and ensures optimum performance regardless of operating conditions. The manufacturer lists the following basic properties as viscosity at 40 ℃ – 68.80 mm²/s, viscosity at 100 ℃ – 10.48 mm²/s, viscosity index – 140 (-) and Total Base Number (TBN) – 10.86 mgKOH/g. Within the lifetime of the engine oil, the manufacturer recommends that the TBN value should be higher than 10.0 mgKOH/g at all times. It also recommends a viscosity range at 100 ℃ of 9.5–12.5 mm²/s. The last limiting value is the maximum age of the engine oil in the internal combustion engine, which is 2 years. The specified engine oil has the following specifications SAE 10 W-30, API CG-4/CF-4/CF/CE/CD/SF, API GL-4, ACEA E3, DIN 51524-3 HVLP.

Based on experience and research on this issue, key physical factors affecting the viscosity of engine oils used in liquid-cooled engines were identified. These include the presence of water, glycol, fuel and soot. Chemical processes that can indirectly affect viscosity include oxidation, nitration and sulphation, as well as changes in the Total Base Number (TBN)^35–38^.

### Methodology for measuring viscosity at 100 ℃

**Fig. 1:**
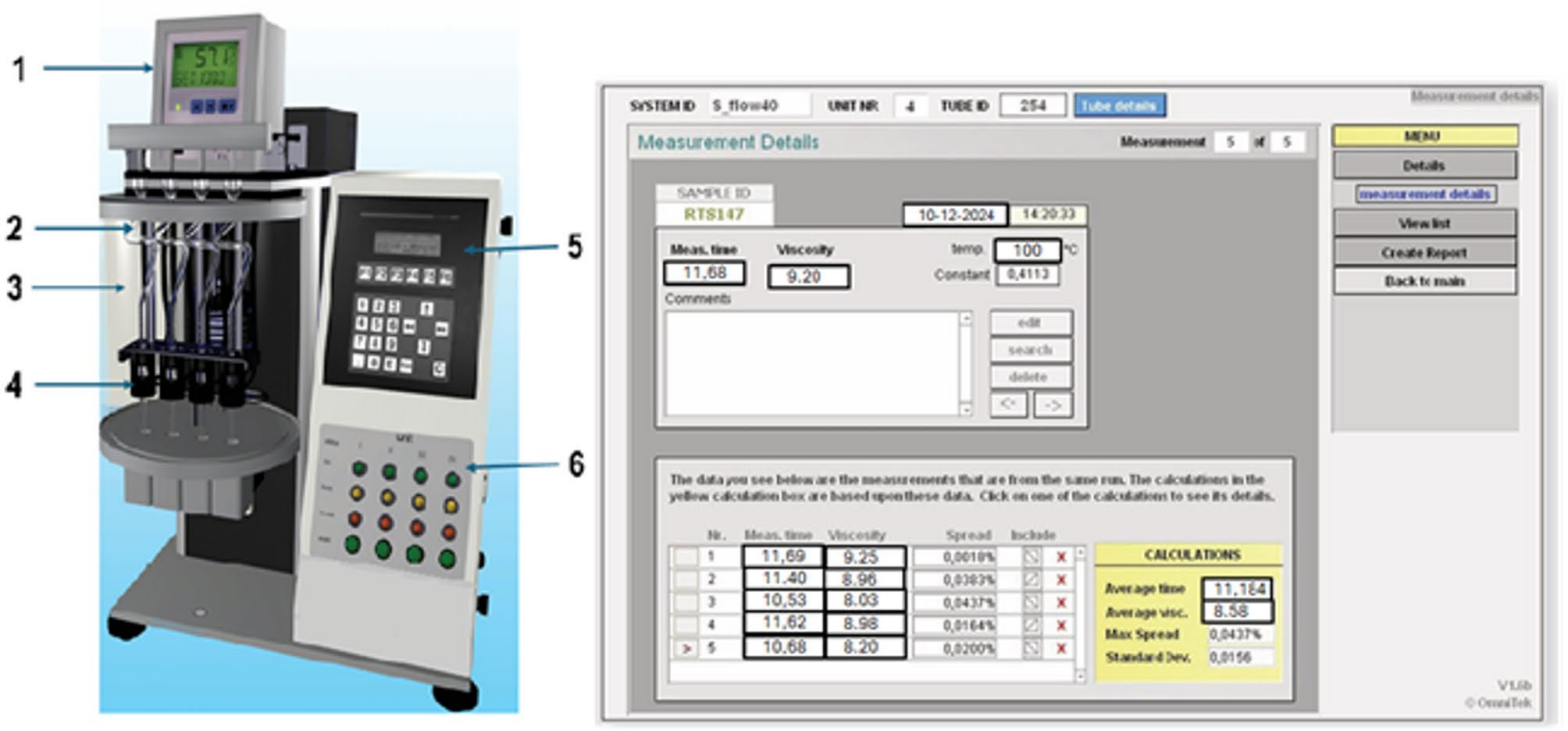
Description of the SPECTRO-Visc Q^300^ and example of the measurement output [own]. Where 1—thermostat, 2—viscometer tubes, 3—thermostatic bath, 4—optical fiber housing, 5—control panel & LCD screen, 6—status LED’s.

The SPECTRO-Visc Q300 (Fig. 1) was used to measure viscosity at 100 ℃, which meets the strict requirements of ASTM D445, D446, D7279, IP 71 and ISO 3104.

**Fig. 2:**
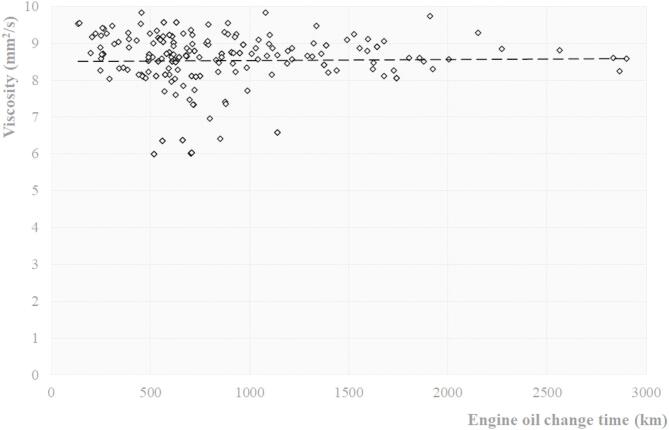
Expression of the dependence of viscosity at 100 ℃ on engine oil change interval [own].

1 $$v=Kt.$$

The kinematic viscosity test is fundamentally based on measuring the flow time of a fixed volume of liquid through the capillary of a calibrated viscometer under a defined hydrostatic pressure and precisely controlled temperature conditions. The kinematic viscosity (*ν*) is determined as the product of the measured flow time (*t*) and the calibration constant (*K*) of the viscometer, where K represents the geometrical characteristics of the instrument^39^. The measured values from the measurements are given in Fig. 2.

### Determination and measurement of elements that affect viscosity in engine oil

Fourier Transform Infrared Spectroscopy (FTIR) is now widely employed for monitoring the thermal and oxidative degradation of engine oils, assessing additive depletion, and detecting contamination in the oil system. Advances in information technology, along with the integration of multidimensional mathematical and statistical tools into spectrometer software, have facilitated the development of predictive models^40,41^. FTIR spectra were pre-processed using baseline correction, smoothing, normalization, and derivatization. These steps reduce noise, remove baseline drifts, and enhance relevant spectral features.

These models enable the derivation of multiple quality parameters of crude oil, lubricants, and fuels from a single spectrum, encompassing both chemical and physical properties. Among the most commonly used multivariate techniques for analyzing entire spectral regions are Principal Component Analysis (PCA) and Partial Least Squares (PLS)^20^ Interval-PLS (iPLS) and Principal Components Regression (PCR)^21^.

Infrared spectrometry is a widely utilized analytical technique primarily intended for the identification and structural characterization of organic compounds. Additionally, it is employed for the detection and quantification of various inorganic substances, including antioxidants, water, soot, oxidation products, nitration, sulfation, and glycol^42^.

**Fig. 3:**
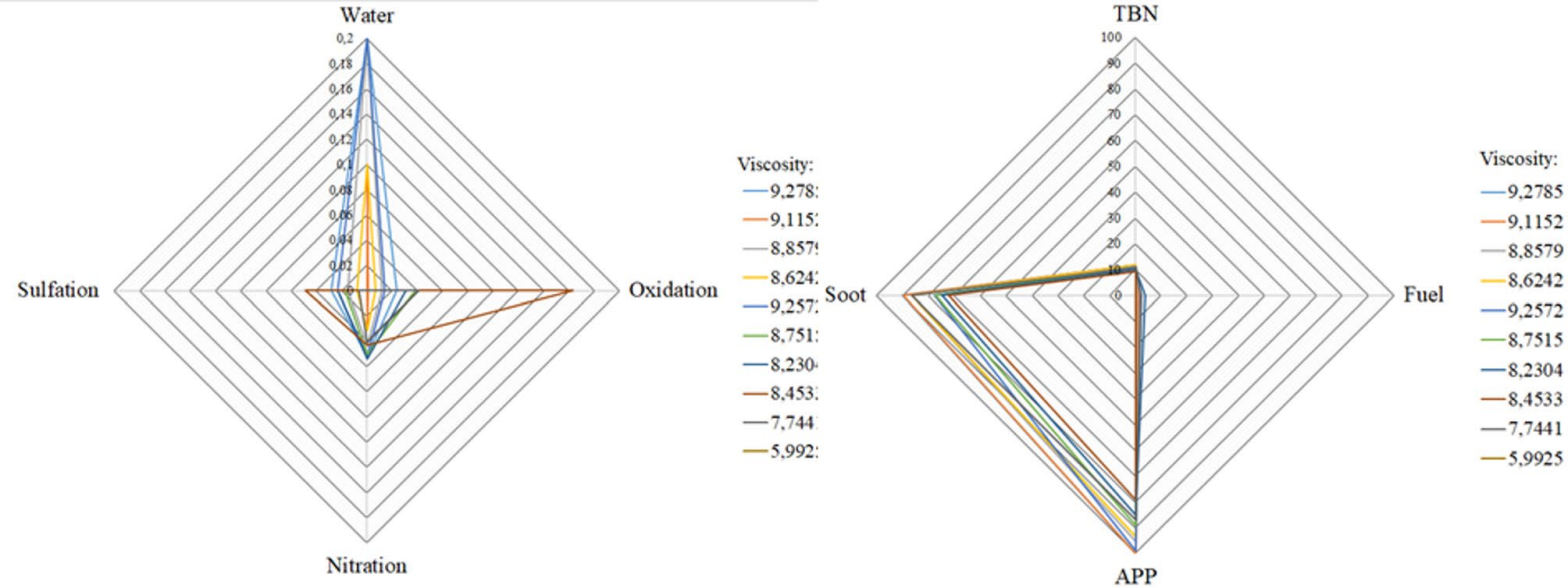
Expression of the dependence of engine oil viscosity on the individual contamination elements [own].

Figure 3 shows the measured values that were obtained in an experiment using dedicated software to Spectro^43^. This software is a key tool in predictive maintenance, contributing to extending equipment life and reducing operating costs.

### Data analysis using multiple linear regression

Multiple linear regression is a statistical method used to model the relationship between a dependent variable (denoted as *y*) and multiple independent variables (denoted as *x*_1_, *x*_2_, …, *x*_p_). It represents an extension of simple linear regression, which involves only one independent variable. This approach enables the analysis of the relationship between a dependent variable (*y*) and multiple independent variables (*x*) and is expressed by the following equation. ^44,45^ Multiple Linear Regression (MLR) was preferred over more advanced machine learning techniques because it provides interpretability and transparency, allowing a clear assessment of each predictor’s contribution. Given the modest dataset size (221 samples), MLR also reduces the risk of overfitting compared to complex models, while still achieving robust predictive performance. Advanced machine learning methods may be considered in future work to further enhance accuracy.

2 $${y_i}={b_0}+{b_1}{x_1}+{b_2}{x_2}+ \ldots \ldots +{b_p}{x_p}+{\varepsilon _i},$$

where $\:{y}_{i}$ – is the dependent variable for each value of kinematic viscosity at 100 °C, $\varepsilon _i$ – is the model error (residual), which includes unexplained variations $\varepsilon _i$~N (0, σ^2^,$\:{\:b}_{0}{,b}_{1},{b}_{2},\:\dots\:,{b}_{p}$ – are the regression coefficients for the influential variables$\:\:{x}_{1},{x}_{2},\:\dots\:,{x}_{\text{p}}$ (in this case, the run in kilometers; TBN number; quantity – fuel, water, glycol, soot, APP (anti-wear particles); number – oxidation, nitration, sulphation; p – denotes the total number of influential variables^45^.

The model error ɛ (residual) expresses the difference between the actual value of the dependent variable y and the value $\hat{y}$ predicted by the model. Then ɛ is expressed as follows

3 $$\epsilon ={y_i} - {\hat {y}_i}$$

where y – the actual value of the dependent variable, $\hat{y}$ – the value of the dependent variable predicted by the regression model.

To assess the performance and goodness of fit of multivariate regression models, four key statistical metrics were employed: Mean Squared Error (MSE), Coefficient of Determination (R²), Akaike Information Criterion (AIC), and Bayesian Information Criterion (BIC).

- **MSE** quantifies the average squared difference between observed and predicted values, being particularly sensitive to large deviations, thus providing a direct measure of model accuracy^46^.
- **R²** represents the proportion of variance in the dependent variable explained by the model, ranging from 0 to 1, reflecting the overall fit of the model^45^.
- **AIC** and **BIC** are information criteria used for model comparison, balancing model accuracy and complexity. Lower values indicate superior models, with BIC imposing a stricter penalty for the number of parameters, offering a nuanced distinction in model selection^45,46^.

BIC is a statistical method used for model selection, similar to the Akaike Information Criterion (AIC), but places more emphasis on penalizing model complexity. A lower BIC value indicates a better model. It takes into account the quality of the fit of the model to the data (logarithm of maximum likelihood) and penalizes models with more parameters more severely.

The main problem with linear regression analysis is the presence of multicollinearity, where the predictor variables are highly correlated with each other. This makes it difficult to unambiguously separate the individual effects of each variable on the dependent variable. As a result, regression coefficients may vary significantly after adding or removing variables. In addition, the standard errors of the regression coefficients increase, reducing the reliability of the estimates and making tests of statistical significance (*p*-values) less reliable.

The Variance Inflation Factor (VIF) is a key statistic in linear regression that is used to assess the degree of multicollinearity between the independent variables in the model. If the VIF exceeds 5, it indicates moderate multicollinearity and requires attention. If the VIF is greater than 10, it indicates a high degree of multicollinearity, which means that the independent variables are highly correlated, which may impair the stability and accuracy of the regression

4 $$VIF=\frac{1}{{1 - R_{k}^{2}}},$$

where $R_{k}^{2}$ – coefficient of determination for regression of the *k*-th independent variable on the other independent variables in the model^47^.

If there are a large number of variables and it is not known which ones are important, it may be useful to first use Stepwise Regression and then apply Bayesian Model Averaging to the selected models. In some cases, overfitting may occur in stepwise regression. Cross-validation was used to mitigate this phenomenon. Regularization methods e.g. LASSO^48^ are recommended for future research.

The Stepwise Regression method is a variable selection technique in regression analysis that combines forward selection and backward elimination methods^49,50^. This procedure automatically selects variables based on statistical criteria such as *p*-value or information criteria (AIC, BIC). *p*-value expresses the probability that the null hypothesis is true, i.e., that adding or removing a given predictor has no statistically significant effect on the explained variable.

The Bayesian Model Averaging (BMA) model is used to select independent variables. The method uses the Bayesian information criterion BIC. A lower BIC indicates a more appropriate model, which is used as a criterion for selecting the best model.

Bayesian Model Averaging (BMA) is a statistical technique designed to address the problem of model uncertainty. In traditional statistical modelling, Backward, Forward and Stepwise methods are used in the process of variable selection in regression models. However, this approach can be problematic because the chosen model may not perfectly capture the true underlying relationship. In fact, there is often considerable uncertainty about which model is optimal, and different models may capture different aspects of the data. Bayesian Model Averaging (BMA) offers an alternative approach. Instead of selecting a single model, it works with all candidate models in the ensemble. BMA weight-averages their predictions, with each model’s weight corresponding to its posterior probability (i.e., the model’s likelihood given the data and prior information)^51,52^. Since there are many possible models, it is difficult to decide which one is “best”. Instead, Bayesian Model Averaging (BMA) assigns a probability to each model based on how well it fits the data. Each model has:

prior probability – reflects our prior knowledge of how likely the model is,

likelihood – how well the model explains the observed data,

posterior probability – is calculated using Bayes’ theorem, which reflects how likely the model is given the data.

Given two models *M*_1_ and *M*_2_ and assuming that one of these models is correct, the posterior probability of the correctness of *M*_*1*_ is as follows^46^

5 $$P({M_1}|y)=\frac{{P(y|{M_1})P({M_1})}}{{P(y|{M_1}) \cdot P({M_1})+P(y|{M_2}) \cdot P({M_2})}}.$$

To compare the two models, the Bayes factor (BF) gives us information about whether the data favors *M*_1_ or *M*_2_

6 $$\frac{{P({M_1}|y)}}{{P({M_2}|y)}}=\frac{{P(y|{M_1})}}{{P(y|{M_2})}} \cdot \frac{{P({M_1})}}{{P({M_2})}}.$$

All analyses were performed in the R language^53^ using mainly the BMA package^51^. The R language is a programming language and environment designed for statistical analysis, data visualization and machine learning. It offers a wide range of statistical methods, from basic descriptive statistics to advanced models (e.g. regression, machine learning, time series, etc.).

## Results and discussion

Using the R programming language, individual calculations were performed to predict the lifetime of the observed engine oil in a diesel internal combustion engine, which has a high power and volume, and is operated under severe conditions. The Pearson correlation coefficient (r) and its associated p-value were used to evaluate linear relationships between variables. Correlations were considered statistically significant at *p* < 0.05 ^54,55^.

**Fig. 4:**
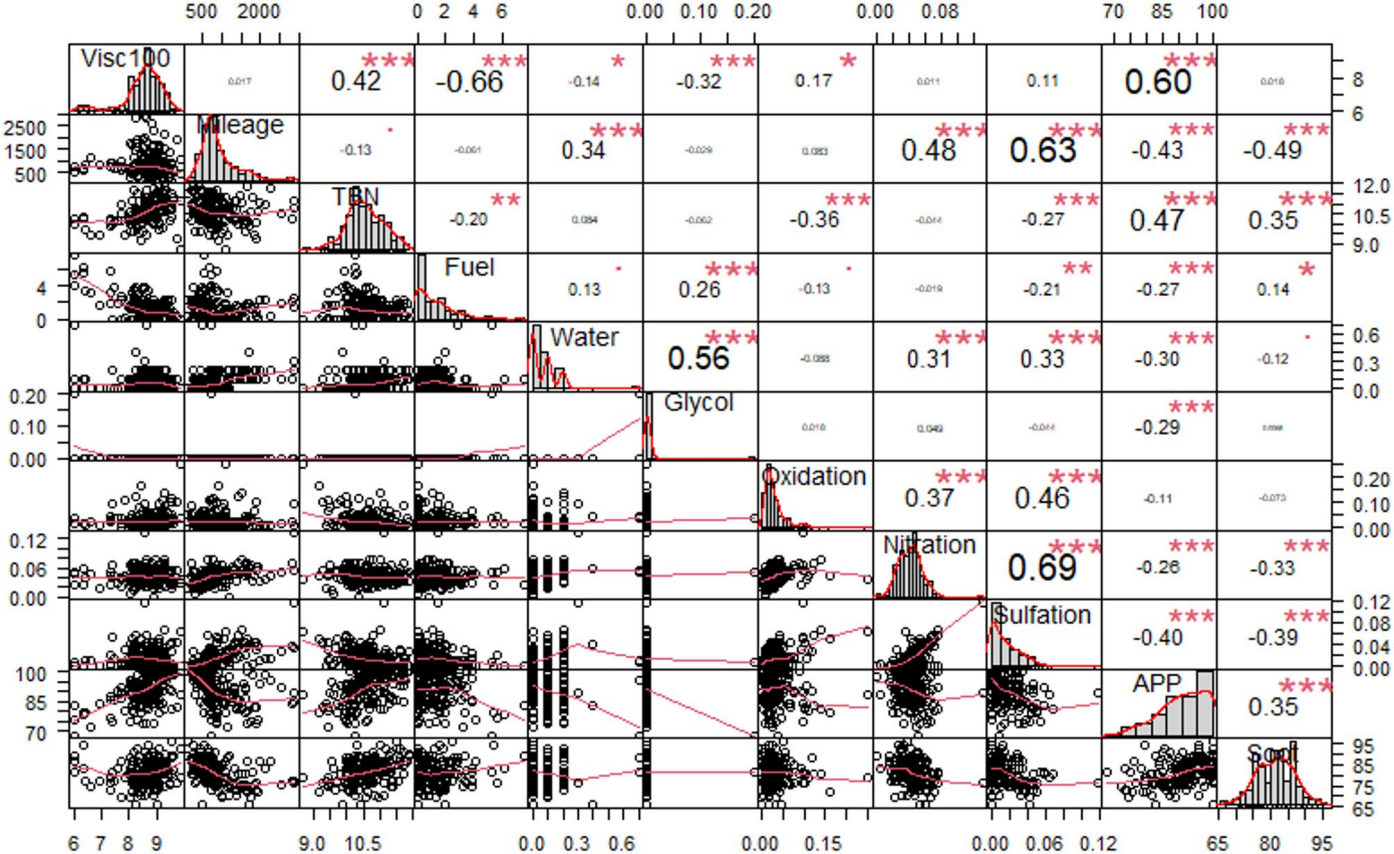
Dependence of the correlation on the calculated values (p, r) between the individual measured parameters of the engine oil. Values *p < 0.05; **p < 0.01; ***p < 0.001 are shown in the table depending on the number of*.

Figure 4 presents the results of Pearson correlation, which assesses the relationship between two variables. The most important is the evaluation of the correlation of Visc100 (viscosity at 100 ℃) with other values. Visc100 correlates strongly with APP (anti-wear particle) with a value of *r* = 0.60. The combination of high temperatures, high load and low viscosity leads to faster consumption of anti-wear additives, resulting in less effective lubrication and increased risk of engine damage^56,57^. The relationship between Visc100 and TBN shows a moderate correlation of *r* = 0.42 (Fig. 4). According to^58^ the TBN level can decrease to a maximum of 65%, at which point an engine oil change is required. In this case, the manufacturer recommends a maximum decrease of 10%. The correlation between Visc100 and oxidation is weak *r* = 0.17 (Fig. 4). The action of air and high temperatures causes oxidation of the oil, the breakdown of its molecules and the formation of varnish deposits. As a consequence, the oil thickens, its kinematic viscosity increases, and replacement is necessary. The oxidation process plays a vital role in affecting viscosity^10,59^. The thickening of lubricating oil due to soot is a major problem in large diesel engines^60^. This leads to an increase in oil viscosity, which reduces its lubricity and affects its passage through the internal combustion engine, especially in cold weather. In our case the value of *r* = 0.01 which shows a very weak correlation.

**Fig. 5:**
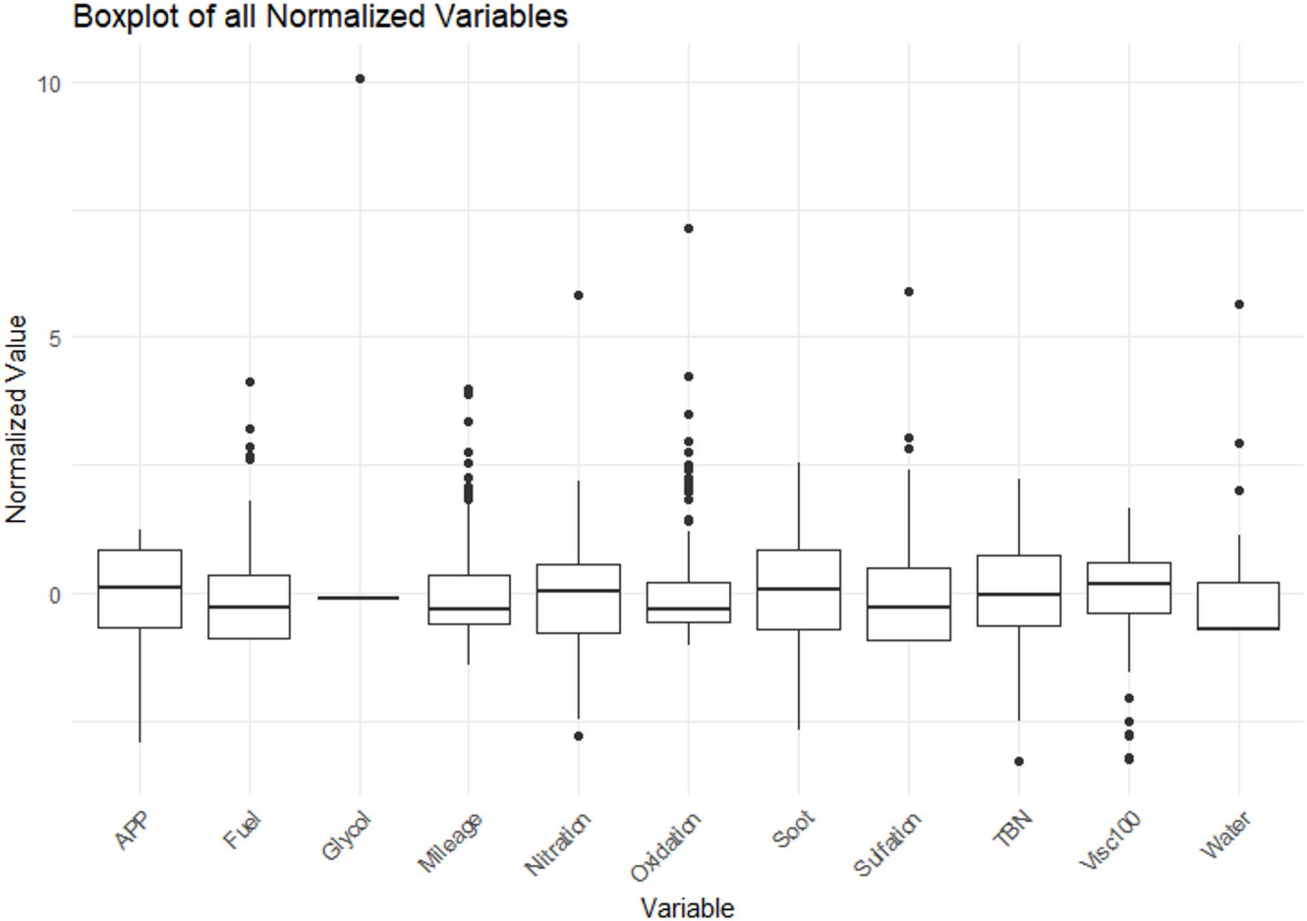
Box plot for normalized data.

The negative correlation between Visc100 and glycol is weak *r* = − 0.32 (Fig. 4). Glycol contamination leads to a significant reduction in the viscosity of the engine oil, which affects its lubricating properties. The reduction may occur due to the breakdown of the molecular structure of the oil due to excessive interaction with glycol, which dilutes the oil and reduces its overall efficiency. The interaction of glycol with the oil causes dilution of the oil and loss of its ability to lubricate properly^61^. The correlation between nitridation and sulfation is moderately strong *r* = 0.69 (Fig. 4). The correlation between nitration and sulfation in engine oil reflects the simultaneous influence of these two chemical processes under severe operating conditions. Both are caused by chemical reactions that take place in the oil due to combustion by-products (NOx and SOx) and are influenced by factors such as temperature, oxidation and fuel composition^62^. Furthermore, according to Fig. 4, a moderately strong negative correlation (*r* = − 0.66) arises between kinematic viscosity at 100 ℃ and fuel contamination by dilution. Too low oil viscosity can result in a thin lubricating film that has low lubricity. The integrity of the lubrication film may be compromised, leading to increased engine wear. This wear is greater when the fuel content of the oil is higher, and the viscosity is lower^5,63^. This can be explained by the fact that fuel has a lower viscosity than engine oil and diluting the oil with fuel will reduce the overall viscosity.

Engine oil analysis data often includes variables with different scales and ranges. Standard Score Normalization allows data to be normalized by converting it to a standard distribution with mean and standard deviation. Normalization helps bring all variables into a common range, increasing consistency and comparability. It also helps in identifying outliers.

(Fig. 5).

- Results of Step-wise.

The process of finding the optimal model was completed with a model including the variables soot, distance, nitration, sulphation, oxidation, fuel, TBN and APP, as this model has the lowest AIC value. AIC = − 357.83 is for the full model (Table 1). The AIC value is used to evaluate the quality of the statistical model. Degree of Freedom expresses the number of degrees of freedom for a given variable in the analysis. In this case, each independent variable has 1 because it represents one estimated parameter in the model. Sum of Squares is the sum of squares that represents the independent variable. This value indicates how much of the explanation of the variability in the dependent variable is accounted for by a particular independent variable. The higher the number, the more it contributes to the variability of the dependent variable. Residual Sum of Squares (RSS) is the residual sum of squares, which expresses the degree to which the model fails to explain the variability of the dependent variable. The higher the RSS number, the less goodness of fit of the model. In the step-wise method, the AIC number serves to select the optimal model by taking into account both the accuracy of the model and its complexity. The lower the number, the better the model. That is, there is a better balance between model accuracy and model complexity.

**Table 1:** Best regression model selection results.

Model AIC = − 357.83				
Variable	Degree of freedom	Sum of Squares	RSS	AIC
Soot	1	0.5389	29.346	−356.20
Mileage	1	10.293	29.836	−352.95
Nitration	1	10.982	29.905	−352.50
Sulfation	1	18.658	30.672	−347.53
Oxidation	1	31.012	31.908	−339.79
Fuel	1	54.477	34.254	−325.88
TBN	1	59.662	34.773	−322.94
APP	1	143.519	43.159	−280.59

(b) A selection of Bayesian models.

**Fig. 6:**
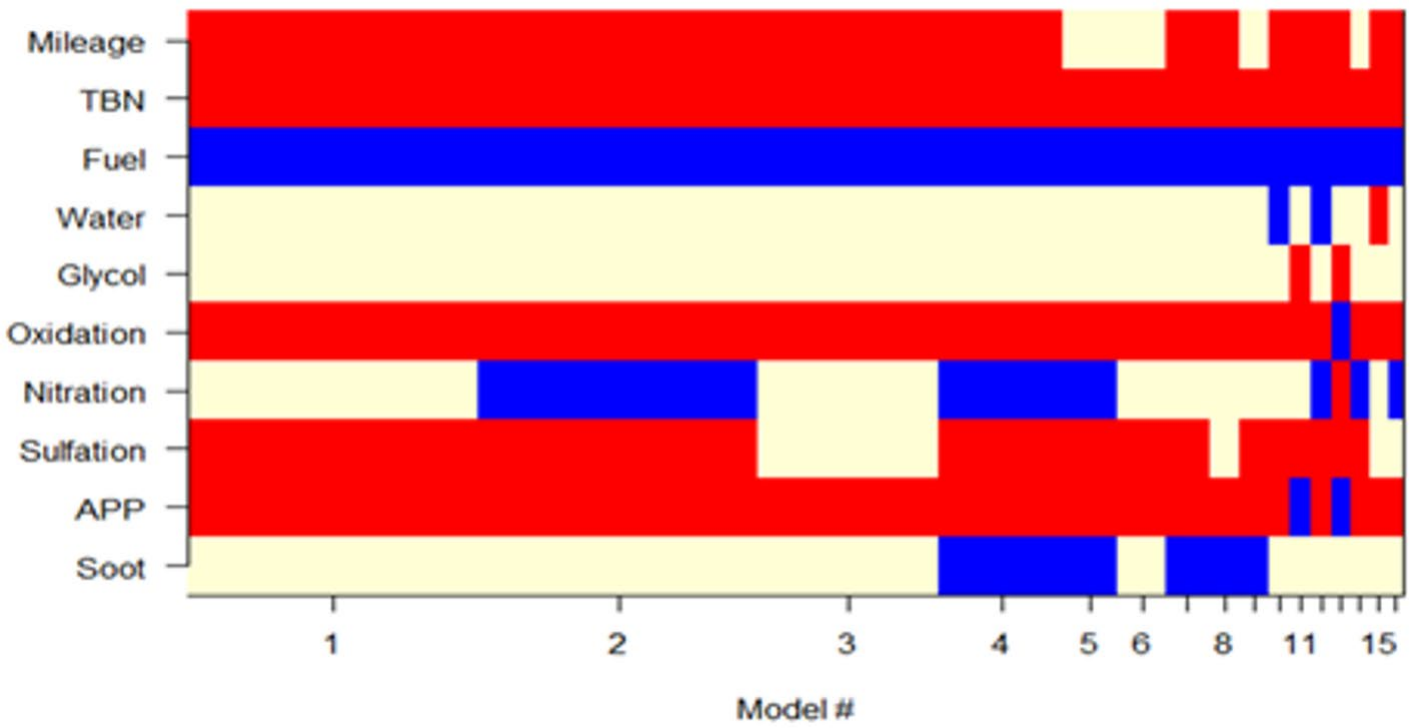
Detailed analysis of the graph “Models selected by BMA”.

The Bayesian models were averaged, and the five best models were created based on the posterior probability values and the lowest BIC values (Fig. 6). From Fig. 6, it can be seen that each row represents an input variable such as distance, TBN, fuel, water, glycol, etc. Furthermore, each column corresponds to a specific model (Model #1 to Model #15). The red colour represents the selected variables in the model and have a positive effect on the dependent variable (Visc100). The blue colour represents the selected variables in the model that have a negative effect on the dependent variable. The yellow colour expresses variables that were not selected in the model, which means they do not have a significant effect or were not included in the model. In addition, Fig. 6 shows the top 15 evaluation models for viscosity prediction. It shows the effects of the variables associated with the BMA models ranked by performance (model 1 is the best). The variables selected for model 1 were mileage, TBN, fuel, oxidation, sulfation, and APP. All variables are statistically significant (Table 2), there is no multicollinearity (Table 3).

Table 2 at the top expresses the regression coefficients fitted to Eq. (16). The bottom row reports the standard errors of estimation for each regression coefficient in the multivariate regression model. Here is the relationship developed for the prediction model for the Visco100 value of engine oil, which can be expressed as follows

7 $$\begin{aligned} Visco(100)&=0.33+0.0002 \cdot Milage+0.340 \cdot TBN - 0.170 \cdot Fuel \hfill \\ & \quad +4.638 \cdot Oxidation+7.073 \cdot Sulfation+0.049 \cdot APP. \hfill \\ \end{aligned}$$

Equation (7) shows that each parameter affects the viscosity of the engine oil in some way. For example, the more miles the vehicle is driven, the more the oil viscosity increases, even if only slightly (coefficient 0.0002). The higher the Total Base Number (TBN), the higher the viscosity (coefficient 0.340). Conversely, a higher amount of fuel in the oil causes a decrease in viscosity (coefficient − 0.170). Oxidation has a significant effect on the increase in viscosity (coefficient 4.638), which means that higher oxidation makes the oil thicker. Sulfation affects viscosity even more than oxidation (coefficient 7.073), which means that the presence of sulfation increases the density of the oil. Conversely, the APP value has less effect on the viscosity of the engine oil (coefficient 0.049). For the statistical evaluation of lubricant viscosity, 60 to 221 samples were analyzed, giving an average viscosity of ≈ 8.54 mm²/s with a standard deviation of ≈ 0.79 mm²/s. This sample range ensures reliable statistical representation at a 95% confidence level.

**Table 2:** Comparison between two models.

Parameters	Dependent variable	
	Visc100	
	(1) Step-wise method	(2) BMA method
Mileage	0.0002** (0.0001)	0.0002*** (0.0001)
TBN	0.429*** (0.069)	0.340*** (0.062)
Fuel	– 0.146*** (0.024)	– 0.170*** (0.023)
Oxidation	6.215*** (1.385)	4.638*** (1.297)
Nitration	– 8.055*** (3.017)	
Sulfation	11.439*** (3.287)	7.073** (2.864)
APP	0.050*** (0.005)	0.049*** (0.005)
Soot	– 0.012* (0.006)	
Constant	0.402 (0.728)	0.330 (0.697)
Observations	196	196
R²:	0.657	0.641
Adjusted R²	0.642	0.630
Residual Std. Error	0.392 (*df* = 187)	0.399 (*df* = 189)
F Statistic	44.772*** (*df* = 8; 187)	56.284***(*df* = 6; 189)

Note: *p < 0.1; **p < 0.05; ***p < 0.01.

Table 3 shows the VIF (Variance Inflation Factor) values for each variable in the Visco100 prediction model. This factor measures multicollinearity between the independent variables.

**Table 3:** Calculated values of variance inflation factor.

Mileage	TBN	Fuel	Oxidation	Sulfation	APP
1.79	1.543	1.18	1.37	2.24	1.84

From the results in Table 3, it can be argued that all the VIF values are less than 5, which means that there is no serious multicollinearity problem among the variables. The highest VIF value is 2.24 (Sulfation), which indicates that this variable is slightly correlated with the others but still within acceptable limits. The lowest VIF value is 1.18 (Fuel), indicating that this variable is almost independent of the others. The degree of multicollinearity in the above model is low, which means that the model is stable and provides reliable predictions.

**Fig. 7:**
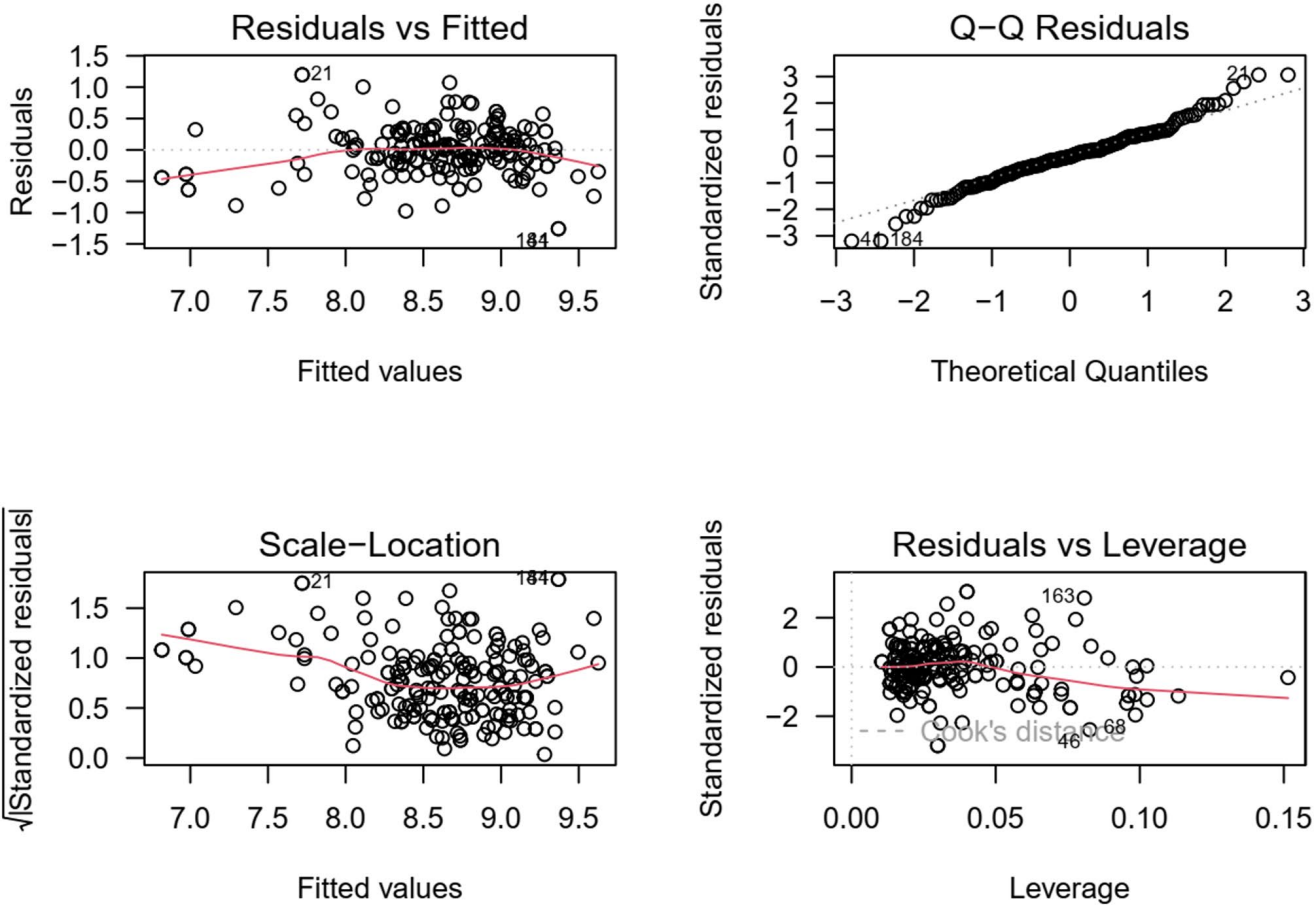
Evaluation of residuals to assess assumptions of multiple linear regression analysis.

In the R model, the residuals (model errors) are analyzed to test the assumptions of multiple linear regression. Key assumptions include linearity, i.e. a linear relationship between the predictors and the dependent variable, which is verified by plotting the residuals against the predicted values. Homoskedasticity implies that the variance of the residuals should be constant, which is diagnosed by plotting the residuals against the predicted values - the residuals should be randomly dispersed with no patterns. A normal distribution of residuals is particularly important for interval estimation and hypothesis testing and is verified by a histogram of residuals or Q-Q plot.

In Fig. 7 shows 4 graphical waveforms that express Residue vs. Fitted (predicted) values. We are looking for a random scatter of residuals around zero with no obvious patterns. In this case, the residuals are randomly dispersed, which means the assumptions of linearity and homoskedasticity are satisfied. The normal Q-Q plot shows how well the residuals follow a normal distribution. In this case, the points are on a straight line, the residuals are approximately normally distributed. Deviations from the straight line (especially at the ends) may indicate a problem with the normal distribution of the residuals. In the Scale-Location plot, the (Spread-Location) plot shows if the residuals are evenly distributed over all levels of predicted values. It looks for a uniform “cloud” of points around a horizontal line. A structured pattern could indicate heteroskedasticity. Residuals vs. Leverage (Cook’s distance) identifies influential points that can have a large impact on the estimates of the regression coefficients. Points with high Cook’s distance values should be examined as possible outliers. Overall, shows that the assumptions of the linear regression model are met.

**Fig. 8:**
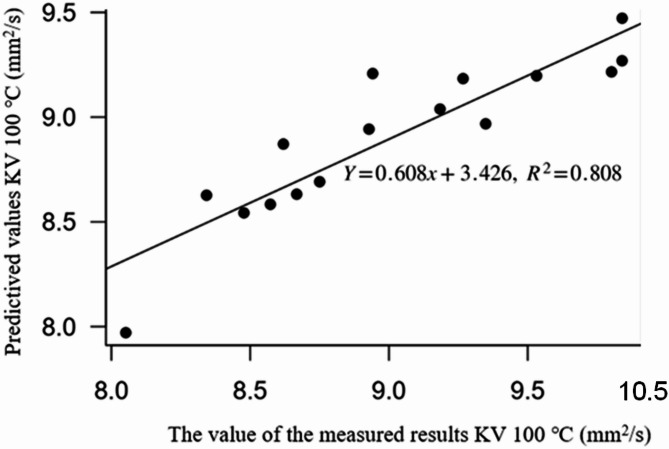
Relationship between observed and predicted values.

Finally, a validation of the calibration model was performed, which is expressed in Fig. 8. Based on formula (8), the value of RMSE (Root Mean Squared Error) is calculated^64^. The evaluation of the prediction model by calibration helps to check how well the model fits the real data and to evaluate the accuracy of the predictions

8 $$RMSE=\sqrt {\frac{1}{n}\sum\limits_{{i=1}}^{n} {{{\left( {{y_i} - {{\hat {y}}_i}} \right)}^2}} } =0.287,$$

where *n –* data points, *y*_*i*_– actual values, ${\hat {y}_i}$ – predicted values.

The RMSE of 0.287 mm²/s represents less than 2% of the operational viscosity range, confirming high practical reliability.

The Fig. 8 shows that the prediction model performs very well for predicting the viscosity at 100 ℃ of engine oil depending on the number of kilometres driven and engine oil degradation parameters such as TBN (Total Base Number), Fuel, Oxidation, Sulfation and APP (anti-wear particle).

In this study, multivariate linear regression (MLR) is combined with Bayesian Model Averaging (BMA) to select suitable variables in predicting engine lubricating oil viscosity. BMA helps to build a stable model by considering the entire model space and taking into account uncertainty.

Compared with other soft computing models (SVM, ANFIS, GPR, MLP) in study^28^ which are prone to overfitting when data is limited and require optimization of many hyperparameters, the MLR model combined with BMA gives stable prediction results, good generalization ability and easier interpretation. Advanced models such as PLS, SVM, and ANN have been discussed but not implemented here due to focus on transparency. Future work will compare MLR with these models.

Our results indicate that the optimal oil replacement time is approximately 200 engine hours that is approximately 2088 km. This estimate is consistent with, and reinforces, the findings of previous studies cited in the article^65^ highlighting the reliability and applicability of this method in predictive maintenance strategies.

## Conclusion

This study confirms that the kinematic viscosity of in-service engine oil at 100 °C can be reliably estimated using a multiple linear regression model based on FTIR-derived parameters. The model incorporates Total Base Number (TBN), fuel dilution, oxidation, sulfation, and anti-wear particle (APP) content as inputs, achieving strong predictive performance (R² = 0.808; RMSE = 0.287). Its main advantage lies in providing a rapid, non-invasive, and cost-effective tool for oil condition monitoring, particularly in field environments where laboratory analyses are limited.

Equation (7) establishes an empirical link between viscosity and FTIR spectra. By applying this relationship, the point of viscosity threshold can be identified and the remaining useful life (RUL) of the lubricant estimated. In the tested heavy-duty diesel engine, the model predicted an oil change interval of ~ 2,088 km (95% CI: 2035–2140 km), consistent with manufacturer guidelines and military field validation.

Sensitivity analysis showed that viscosity changes are driven mainly by TBN depletion, fuel dilution, soot accumulation, nitridation, sulfation, and APP content. Focusing on these markers enables efficient lubricant quality assessment and supports condition-based maintenance (CBM) strategies for improved engine reliability and operational readiness.

However, the model has limitations. It was developed for a specific engine type and operational profile, and its generalizability to other engines, fuels, or duty cycles remains uncertain. Future studies should validate the approach on broader datasets and explore advanced machine learning methods (e.g., random forests, gradient boosting, neural networks) to further improve accuracy and robustness.

In conclusion, the presented regression model provides a practical and mathematically grounded framework for predicting oil viscosity from targeted FTIR data. Its integration into maintenance planning can optimize oil change intervals, reduce unnecessary lubricant replacement, and enhance predictive maintenance practices in both military and civilian heavy-duty applications.

## Data Availability

All data generated or analyzed during this study are included in this published article.

## Abbreviations

FTIR: Fourier transform infrared spectroscopy
TBN: Total base number
APP: Anti-wear particles
BMA: Bayesian model averaging
RMSE: Root mean squared error
SDGs: Sustainable development goals
VI: Viscosity index
TAN: Total acid number
PPR: Projection pursuit regression
PLS: Partial least squares
SVM: Support vector machines
MAE: Mean absolute error
MSE: Mean squared error
GLM: General linear model
PCA: Principal component analysis
PCR: Principal component regression
RUL: Remaining useful life
SAE: Society of automotive engineers
API: American Petroleum Institute
ACEA: Association des Constructeurs Européens d’Automobiles
DIN: Deutsches Institut für Normung
HVLP: High Viscosity Index, Low Pour point
ASTM: American Society for Testing and Materials
R^2^: Coefficient of determination
SS_res_: Residual sum of squares
SS_tot_: Total sum of squares
AIC: Akaike Information Criterion
BIC: Bayesian Information Criterion
VIF: Variance Inflation Factor
